# Multilevel non-contiguous thoracic pedicle subtraction osteotomy for fixed rounded hyperkyphotic deformity of the thoraco-lumbar junction with anterior bony fusion: technical note

**DOI:** 10.1186/s10195-022-00665-4

**Published:** 2022-09-19

**Authors:** Cesare Faldini, Francesca Barile, Giovanni Viroli, Marco Manzetti, Giuseppe Geraci, Alberto Ruffilli

**Affiliations:** Department of Biomedical and Neuromotor Science—DIBINEM, University of Bologna, 1St Orthopaedic and Traumatologic Clinic, IRCCS Istituto Ortopedico Rizzoli, Bologna, Italy

**Keywords:** Kyphosis, Thoracolumbar junction, Pedicle substraction osteotomy, Sagittal imbalance

## Abstract

**Background:**

Fixed severe hyperkyphotic deformities spread over more than five vertebral levels represent a therapeutic challenge, especially when the deformity apex is located at the thoraco-lumbar junction, thus requiring a huge amount of correction. The aim of this article is to describe an innovative all-posterior corrective technique based on multilevel non-contiguous thoracic pedicle subtraction ostoeotomy (PSO).

**Materials and methods:**

A retrospective review of three patients with fixed severe thoracic hyperkyphosis (a deformity angle of over 70°) with a thoraco-lumbar apex (between T11 and L1) treated by simultaneous two-level thoracic PSO and thoraco-lumbar posterior fusion was performed. Radiographic and clinical records were evaluated pre-operatively, post-operatively and at last follow-up (after a minimum of 2 years). Each variable was presented as mean ± SD (standard deviation). Statistical analyses were performed using paired* t*-tests (*P* value < 0.05 was considered significant).

**Results:**

The mean local deformity angle decreased by 75% (from 81.3° ± 2.1° to 20.7° ± 1.4°, *p* < 0.001), the post-operative thoracic kyphosis decreased by 46% (from 61.4° ± 2.4° to 33.2° ± 0.9°, *p* < 0.001) and the sagittal vertical axis decreased by 73% (from 14.7 cm ± 0.8 cm to 3.9 cm ± 0.3 cm, *p* < 0.001). No differences were observed in the radiological results between post-operative values and those at the final follow-up. The average Oswestry Disability Index (ODI) score reduced from 65.7 ± 1.8 pre-operatively to 17.3 ± 1.7 at last follow-up (*p* < 0.001). No neurological, mechanical nor infective complication occurred.

**Conclusions:**

The presented technique, although technically demanding, proved to be a safe and effective alternative for the management of fixed severe thoraco-lumbar junction hyperkyphotic deformities.

*Level of evidence:* IV

*TRIAL REGISTRATION* Retrospectively registered

## Introduction

Fixed severe hyperkyphotic deformities spread over more than five vertebral levels are complex to treat due to the amount of correction required, which varies depending upon the angular value and the localization of the apex of the deformity. The most challenging situation is when the apex of the kyphosis is located at the thoraco-lumbar junction because the maximal correction is required at this site [1].

In the presence of severe deformities, the huge amount of angular correction, over 70°, that is needed remains complex to obtain, even by performing aggressive tricolumnar osteotomies [2]. Pedicle subtraction osteotomy (PSO) and bone disc bone osteotomy (BDBO) are capable of guaranteeing maximal angular corrections of 35° and 55°, respectively [3]. Vertebral column resection (VCR) represents the most powerful spinal osteotomy; it allows tremendous corrective angles but encompasses a high rate of neurological and mechanical complications and should therefore be reserved for biplanar severe fixed deformities in which the spine must not only be shortened but also translated [4].

The authors have therefore speculated that the treatment of these severe deformities should be accomplished by means of two simultaneous PSOs performed non-contiguously, permitting adequate corrective potential alongside excellent intraoperative control and healing potential of the osteotomy sites, thus resulting in a low rate of neurological and mechanical complications.

The aim of this article is to describe a posterior one-stage technique based on multilevel non-contiguous thoracic PSO for severe (a deformity angle of over 70°) fixed rounded kyphotic deformities at the thoraco-lumbar junction, including the correction and complication rates at a minimum of 2 years’ follow-up in three adult patients.

## Materials and methods

### Study sample

A retrospective review of patients over 18 years old with fixed severe thoracic hyperkyphosis (a deformity angle of over 70°) with a thoraco-lumbar apex (between T11 and L1) treated by simultaneous two-level thoracic PSO and thoraco-lumbar posterior fusion was performed. Follow-up evaluations were performed post-operatively, at 12 months and up to the final follow-up at 28.7 ± 2.5 months.

Informed consents for participation in the study and for the publication of clinical images were obtained from each patient.

### Data collection

Included patients were all affected by severe fixed thoracic hyperkyphosis with a thoraco-lumbar apex (between T11 and L1). Anterior bony fusion at the apex of the deformity was present in each case and documented by pre-operative CT scan. Fulcrum supine radiographs were taken in all cases to confirm the irreducibility of the deformity.

Patient demographics, the aetiology of the deformity and data on prior surgical treatments were collected by reviewing the medical records.

Operative time, blood loss, length of stay, and intra- and post-operative complications were recorded.

The deformity angle (used to evaluate the local spinal kyphosis angle, defined as the Cobb angle from the upper endplate of the proximal junctional normal vertebra to the lower endplate of the distal junctional normal vertebra) and the T1–T12 thoracic kyphosis (TK) and L1–S1 lumbar lordosis (LL) angles were measured on pre- and post-operative full-length standing radiographs. The C7 plumbline (C7PL)/central sacral vertical line (CSVL) and sagittal vertical axis (SVA) were used to assess coronal and sagittal imbalance. The pelvic incidence (PI), pelvic tilt (PT), sacral slope (SS), PI–LL mismatch (PI-LL) and GAP score [5] were used to evaluate the spinopelvic balance.

The sagittal deformity angular ratio (S-DAR) [6] was used pre-operatively to evaluate the radius of curvature of the deformity, and was calculated by dividing the deformity angle by the number of vertebral bodies in the deformed area.

Osteotomy healing was assessed in all cases with a CT scan performed at the 1-year follow-up evaluation.

The Oswestry Disability Index (ODI) was administered pre-operatively and at last follow-up.

### Surgical planning and technique

Surgery was planned based on pre-operative full standing antero-posterior and lateral X-rays of the entire column and on CT scan; these guided the decision regarding the location of the osteotomies and the desired corrective angle, the fusion area, and eventually the need to obtain patient-specific guides for screw insertion.

The first osteotomy was always planned at the apex of the deformity, while the second was always planned at three levels above in order to maintain at least three pairs of screws between the two osteotomies. The degree of correction for each osteotomy was accurately measured on the 3D CT scan reconstruction.

**Fig. 1:**
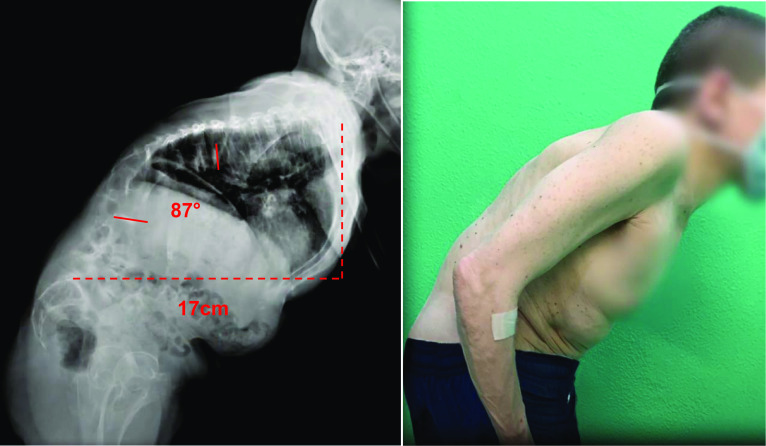
A 54-year-old female (case 1) with severe iatrogenic thoraco-lumbar hyperkyphosis (prior uninstrumented anterior and posterior thoraco-lumbar fusion): pre-operative radiographic and clinical presentation

Regarding the fusion area, the lower instrumented vertebra (LIV) was the sagittal stable vertebra according to the definition by Cho et al. [7]. The fusion was eventually extended distally in the presence of documented disc degeneration at the level below the planned LIV (Fig. 1). The upper instrumented vertebra was T3 in all cases.

**Fig. 2:**
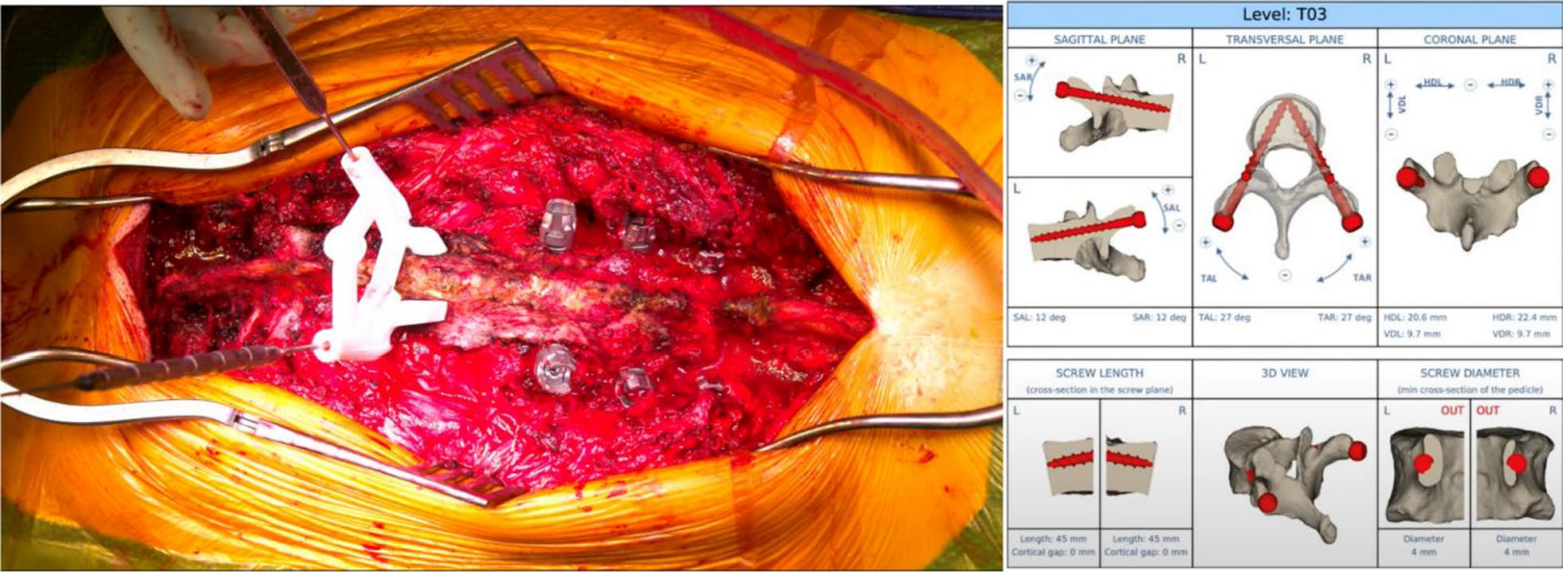
Patient-specific guides were used for pedicle screw placement because of the abnormal anatomy of posterior elements due to prior fusion

In the presence of an altered posterior anatomy (related to previous surgeries) with the presence of posterior spinal fusion mass, customized guides (Myspine, Medacta International SA, Castel San Pietro, Switzerland) for the placement pedicle screws were developed (Fig. 2); otherwise, the freehand technique was used.

The patient was placed prone on an Allen table; neurophysiologic monitoring with somatosensory-evoked potentials and trans-cranial motor-evoked potentials was used in all cases. After a standard posterior midline approach with longitudinal skin incision, careful subperiosteal exposure of the spinous processes and laminae was performed; when customized guides were used (in two cases), care was taken not to remove bone to avoid altering the contact points of the guides. Each guide was then placed on the corresponding vertebra and firmly held; then, after checking the contact surfaces, lateral and contralateral awls were used to prepare the entry point, and a 2.5 mm drill was used to prepare the pathway for the screws in the pedicle [8]. Finally, after tapping, polyaxial screws of an appropriate length and diameter were inserted according to the planned model. When customized guides were not needed (in one case), a freehand technique was used [9].

Screws were placed at all levels, except for those in planned osteotomies. The screw position was checked with a fluoroscope.

**Fig. 3:**
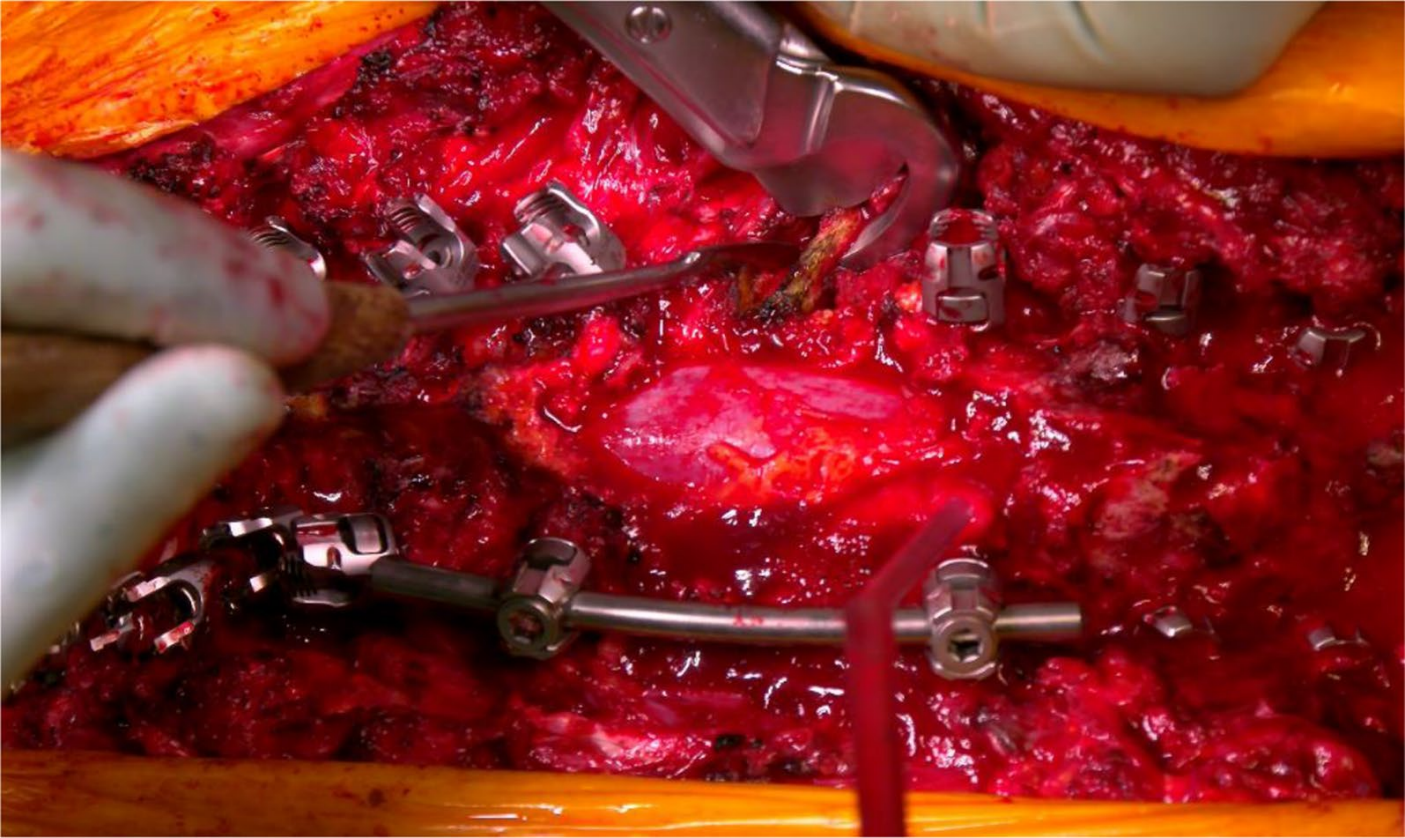
Wide laminectomy, temporary rod placement and rib resection at T12

Then, the first thoracic PSO was performed as planned, at the apex of the deformity (T12 in two cases, T11 in one case). Laminectomy was performed at the level of the osteotomy and at the adjacent levels, one above and one below, to prevent buckling of the spinal cord during the correction manoeuvre; the pedicles were visualized and drilled to maintain orientation. The ribs at the level of the osteotomy were carefully exposed (3–4 cm) and removed, disarticulating the costotransverse joints bilaterally (Fig. 3). The bone was all saved for later fusion. Then, careful dissection of the lateral vertebral body wall was carried out bilaterally.

**Fig. 4:**
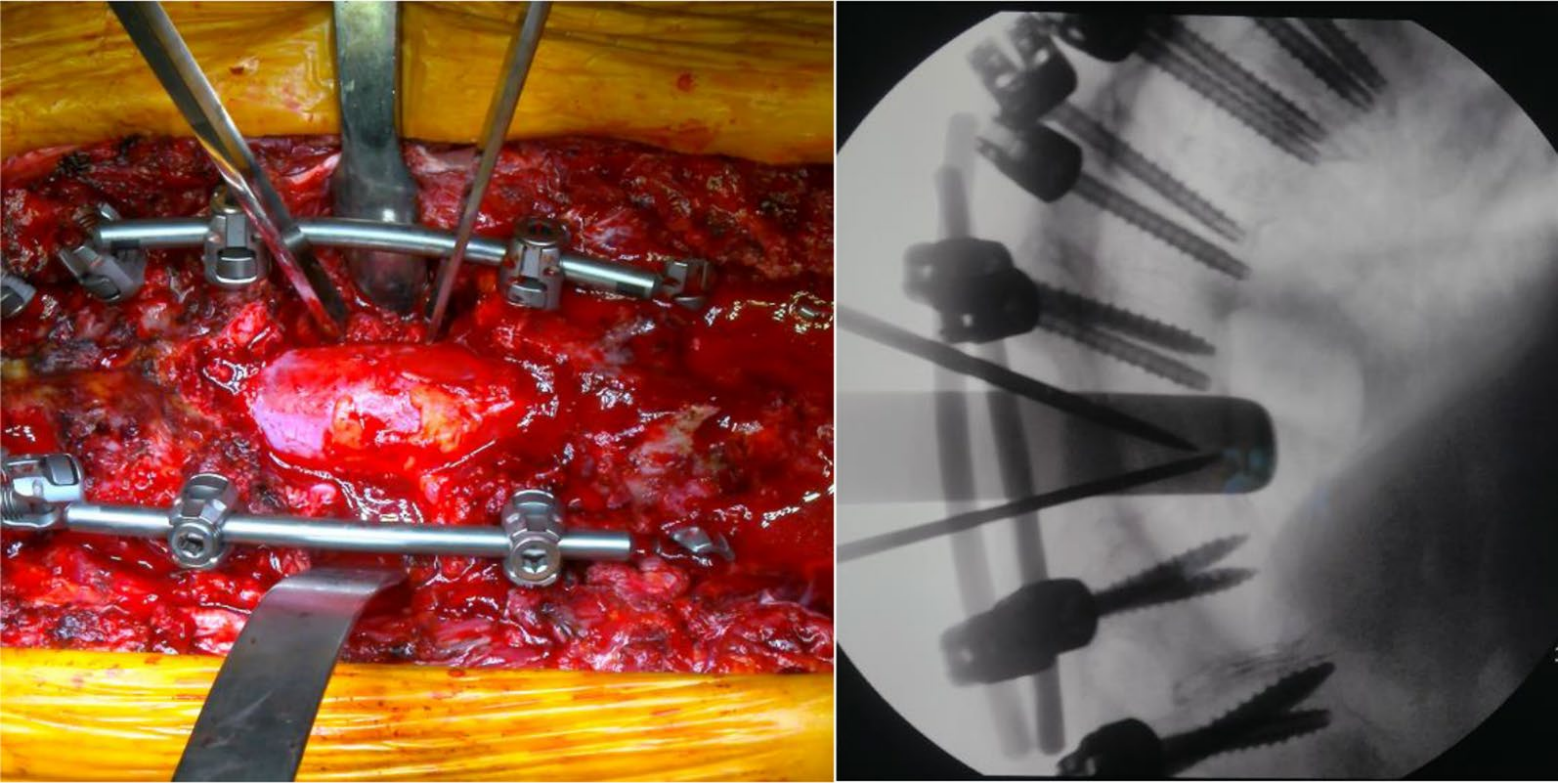
Surgical and radiographic pictures of the first PSO, performed at T12 using a bone scalpel

After positioning dural retractors to optimize the visualization of the vertebral body, two osteotomes were positioned superior and inferior with respect to the pedicle, and the desired osteotomy angle was checked under fluoroscope guidance (Fig. 4). A temporary rod was placed on the opposite side to the first surgeon to prevent sudden collapsing of the spine and possible translation while completing the osteotomy. Then, lateral vertebral body wall cuts were made with straight osteotomes in a precise wedge according to the desired degree of closure.

**Fig. 5:**
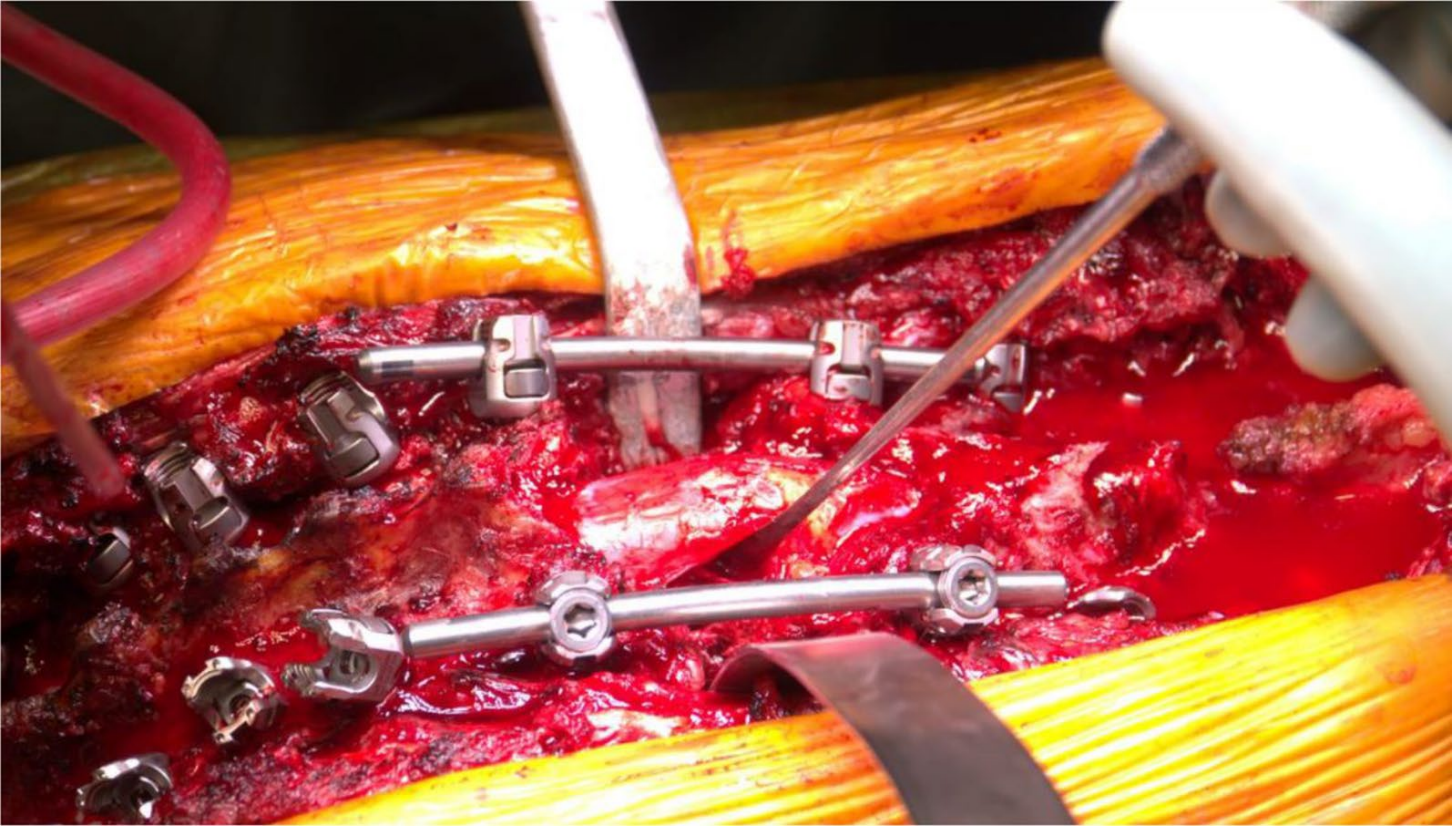
Careful assessment of the complete posterior wall resection in order to avoid dural sac entrapment during the subsequent osteotomy closure

The apex of the wedge was at the cortical anterior vertebral body wall, which was carefully violated with a sharp osteotome to obtain the desired corrective angle. The bony wedge was then removed. The same procedure was performed on the opposite side. Thoracic roots were preserved in all cases. All soft tissues and osseous elements were carefully removed from the anterior portion of the dura to prevent any compression of the neural elements anteriorly during osteotomy closure (Fig. 5).

PSO closure was then performed by gentle compression across temporary rods and by adjusting the Allen bed frame to reduce the kyphosis.

**Fig. 6:**
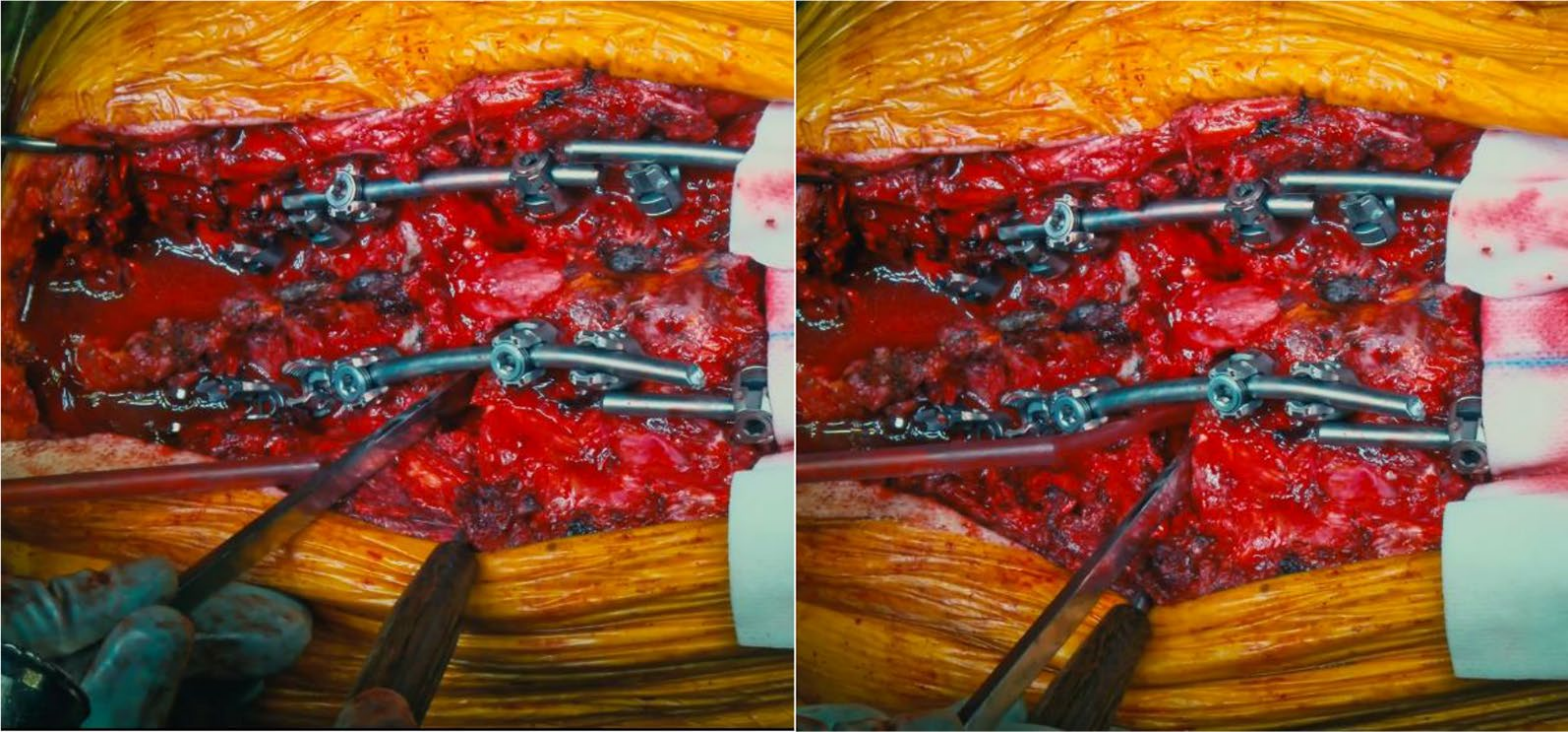
The second PSO is performed at T8

**Fig. 7:**
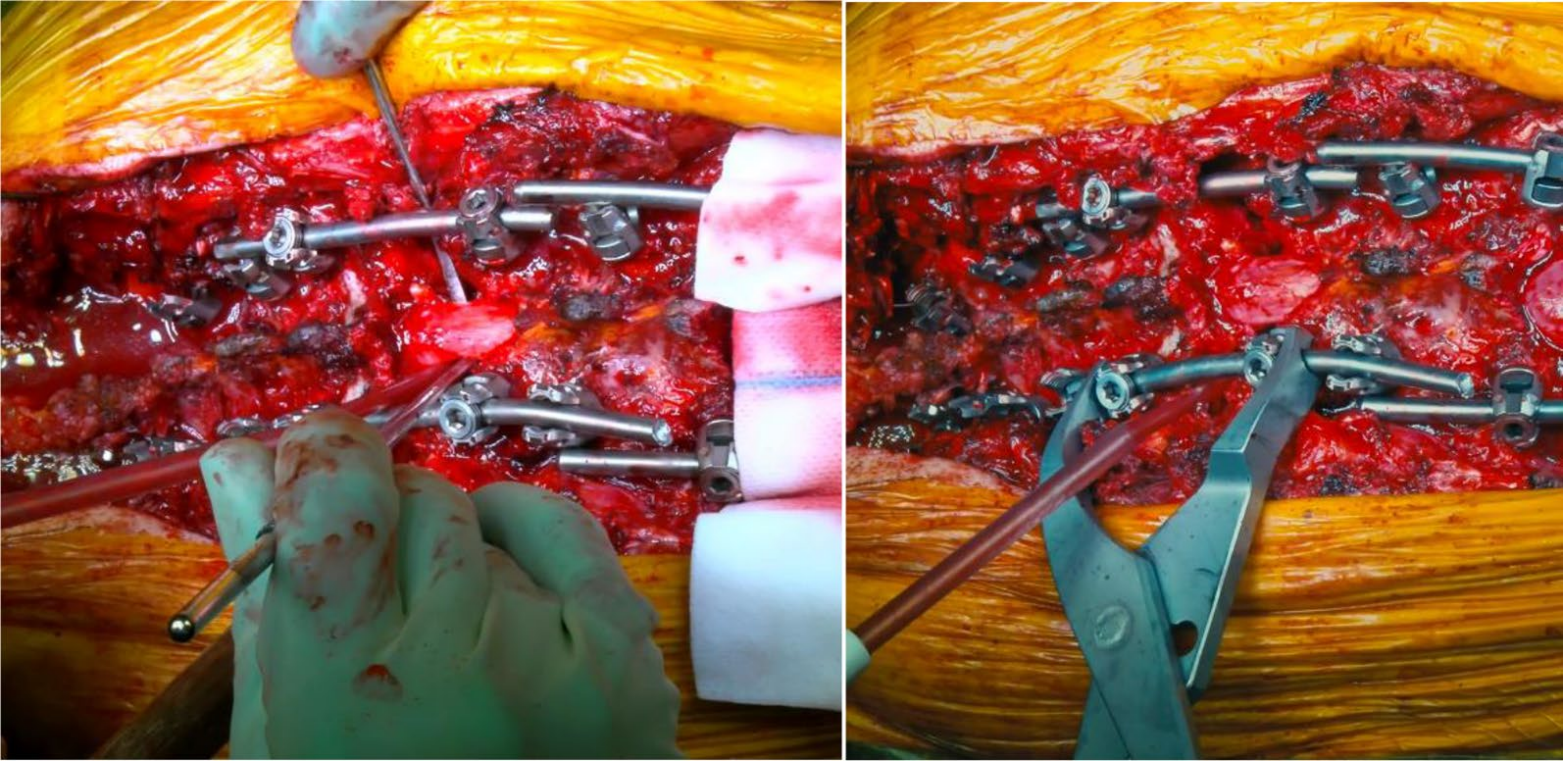
Careful assessment of the complete posterior wall resection and subsequent osteotomy closure

The same procedure was repeated on the other thoracic vertebra (T8 in two cases, T7 in one case) to achieve proper correction of the deformity (Figs. 6, 7).

**Fig. 8:**
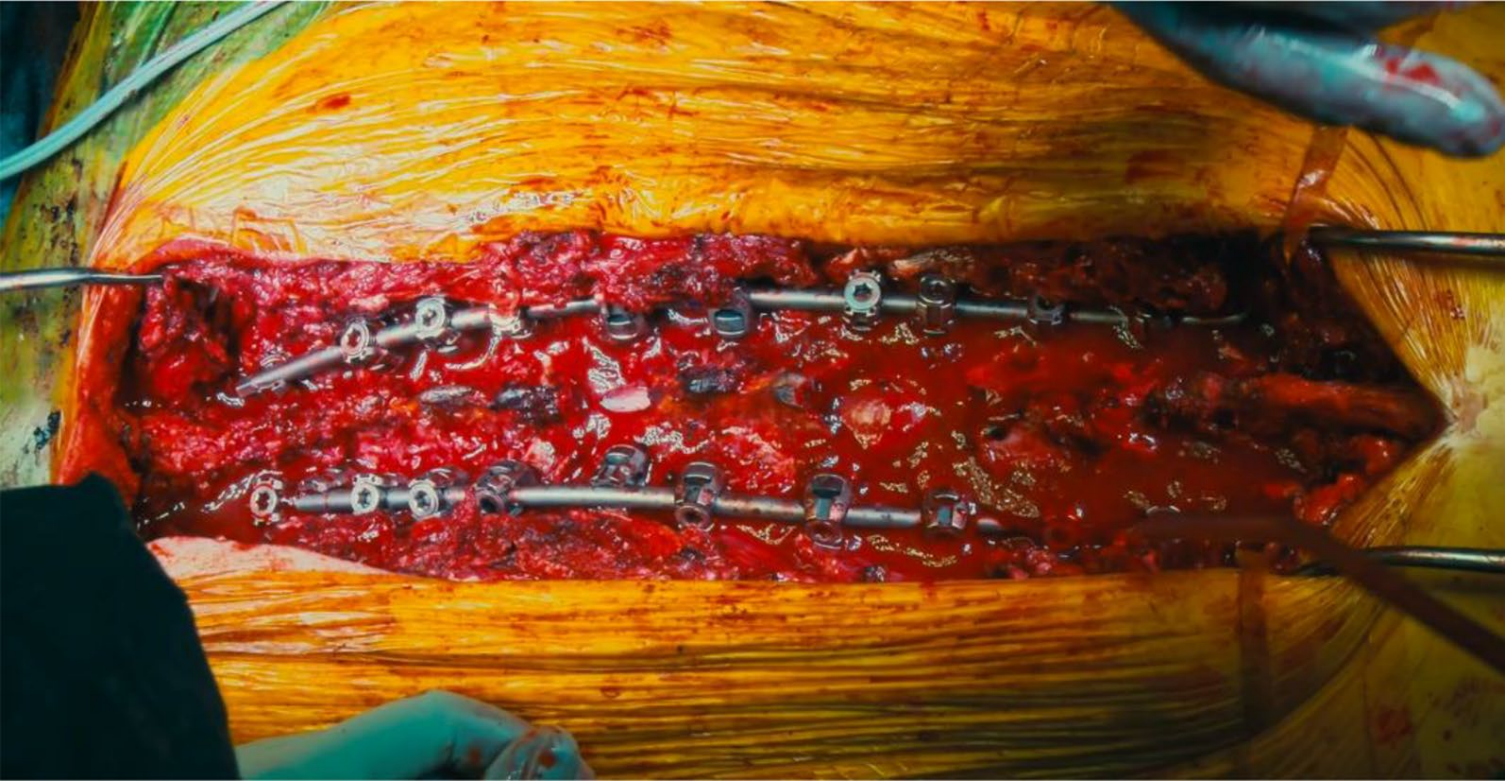
Definitive rods are placed after both osteotomies are closed

Then, the temporary rods on the right side were removed and gently replaced with a pre-bent 5.5 mm titanium rod; the same procedure was performed on the left side. After placing the definitive rods (Fig. 8), segmentary compression was applied to the screws and rods to achieve bone-to-bone closure.

**Fig. 9:**
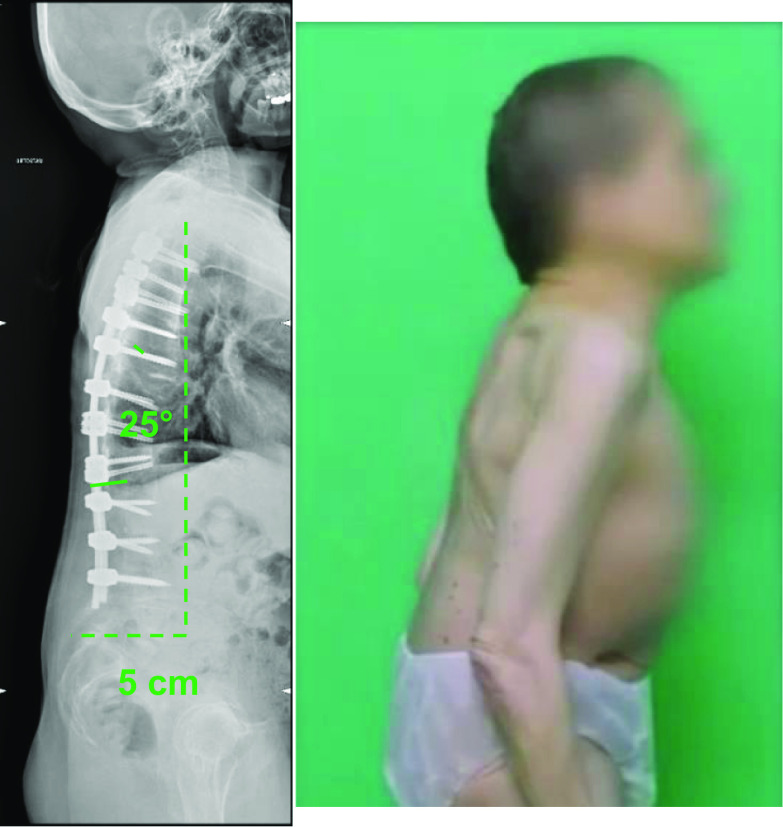
Case 1: postoperative radiographic and clinical presentation

Careful decortication of the posterior spinal elements of the instrumented vertebrae was followed by apposition of allograft bone. A subfascial (Fig. 9) drain was placed and a standard suture was performed.

Early mobilization out of bed started on post-operative day 1. For the first 12 weeks after surgery, a thoracic lumbar sacral orthosis (TLSO) to restrict spinal movements and facilitate initial bone graft fusion was prescribed.

### Patient characteristics

Three patients (two females and one male) were included. The average age was 42.6 (range 30–52) years. Patient characteristics are summarized in Table 1. According to PI, the Roussouly morphotype of all patients was 4 [10].

**Table 1:** Patient characteristics

Patients *n*	3 (1 male, 2 females)
Average age (years)	42.6 (range 30–52)
Curve etiology	Iatrogenic in 2 cases, congenital in 1 case
S-DAR	28.4 ± 3.3
Deformity apex level	T12 in 2 cases, T11 in 1cases
Screw insertion technique	Patient-specific guides in 2 cases (iatrogenic deformities) and the freehand technique in 1 case
Average fused levels	13.3 ± 0.6
Average pedicle screws implanted	22 ± 1.7
Screw density	0.96 ± 0.02
Level of the osteotomies	T8 and T12 in 2 cases, T11 and T7 in 1 case
Average surgical time (min)	305 ± 56
Average blood loss (mL)	1982 ± 976
Average length of stay (days)	9.7 ± 3.1
Average follow-up (months)	28.7 ± 2.5

### Statistical analysis

Each variable was presented as mean ± SD (standard deviation). Statistical analyses were performed using paired* t*-tests (SPSS version 17.0). Normality was assumed, and a* P* value < 0.05 was considered significant.

## Results

The mean operating time was 305 ± 56 min, and the mean blood loss was 1982 ± 976 ml. Intra-operatively, the surgical planning regarding the fusion area was respected in all the cases; there was no need to extend the instrumentation proximally or distally due to the pulling out of screws during a corrective manoeuvre at the most cephalad and caudal levels. Intra-operatively, an accidental durotomy was observed in one case during the laminectomy at the T12 level, with a transient reduction of somatosensory-evoked potentials and trans-cranial motor-evoked potentials in both lower limbs. The dural lesion was sutured and sealed with fibrin glue. Somatosensory-evoked potentials and trans-cranial motor-evoked potentials spontaneously returned to baseline values in approximately 25 min with the administration of steroids and blood products, and the procedure was completed. No reduction in somatosensory- and motor-evoked potentials was observed during osteotomy closure in any of the cases. All the patients were neurologically intact after surgery. Patients were discharged after an average length of stay of 9.7 ± 3.1 days. Significant results were achieved in the sagittal plane (Table 2). In detail, the mean local deformity angle decreased by 75% (from 81.3° ± 2.1° to 20.7° ± 1.4°, *p* < 0.001), the post-operative TK decreased by 46% (from 61.4° ± 2.4° to 33.2° ± 0.9°, *p* < 0.01) and the sagittal vertical axis decreased by 73% (from 14.7 cm ± 0.8 cm to 3.9 cm ± 0.3 cm, *p* < 0.01). No significant change was noted in spinopelvic parameters; however, the GAP score showed a significant improvement (from 10.7 ± 1.5 to 7.3 ± 1.5, *p* < 0.05).

**Table 2:** Average values of the examined variables pre-operatively, post-operatively, and at the 1-year and 2-year follow-up

	Pre-op	Post-op	% variation pre-op vs post-op	*P*-value pre-op vs post-op	1-year FU	Last FU	% variation pre-op vs last FU	*P*-value pre-op vs last FU	% variation post-op vs last FU	*P*-value post-op vs last FU
DA	81.3° ± 2.1	20.7° ± 1.4	−75%	< 0.001*	20.8° ± 1.3	20.8° ± 1.4	−74%	< 0.001*	1%	0.33
TK	61.4° ± 2.4	33.2° ± 0.9	−46%	< 0.01*	33.1° ± 0.9	32.9° ± 0.8	−46%	< 0.01*	−1%	0.24
LL	51.3° ± 2.1	48.2° ± 1.3	−6%	0.07	48.1 ± 1.2	48.4° ± 1.1	5%	0.13	1%	0.41
PI	85.8° ± 2.3	85.9° ± 2.2			85.8° ± 2.1	85.8° ± 2.2				
PT	45.3° ± 4.1	46.6° ± 2.5	+ 2.8%	0.92	46.3° ± 2.7	45.9° ± 2.6	+ 1.3%	0.93	−0.8%	0.87
SS	40.6° ± 5.9	39.3° ± 4.8	−3.2%	0.92	39.6° ± 4.6	39.9° ± 4.7	−1.7%	0.93	+ 0.7%	0.88
PI-LL	34.9° ± 3.2	37.9° ± 2.9	+ 8.5%	0.42	37.9° ± 2.9	37.4° ± 2.8	+ 7.1%	0.38	−1.3%	0.77
GAP score	10.7 ± 1.5	7.3 ± 1.5	−31.7%	< 0.05*						
C7PL/CSVL	2.9 cm ± 1.0	1.4 cm ± 0.2	−46%	0.15	1.3 cm ± 0.3	1.1 mm ± 0.2	−55%	0.12	−19%	0.07
SVA	14.7 cm ± 0.8	3.9 cm ± 0.3	−73%	< 0.01*	3.8 cm ± 0.3	3.7 cm ± 0.2	−75%	< 0.01*	−5%	0.55
ODI	65.7 ± 1.8					17.3 ± 1.7	−74%	< 0.01*		

*FU* follow-up, *DA* deformity angle, *TK* thoracic kyphosis, *LL* lumbar lordosis, *PI* pelvic incidence, *PT* pelvic tilt, *SS* sacral slope, *PI-LL* Pelvic incidence–lumbar lordosis mismatch, *C7PL/CSVL* C7 plumb-line (C7PL)/central sacral vertical line (CSVL), *SVA* sagittal vertical axis, *ODI* Oswestry Disability Index

^*^statistically significant

The average C7PL/CSVL remained at a physiological value of 1.4 cm ± 0.2 cm; there was no difference with respect to the pre-operative value (n.s.).

No differences were observed in the radiological results between post-operative values and those obtained at final follow-up.

The average Oswestry Disability Index (ODI) score reduced from 65.7 ± 1.8 pre-operatively to 17.3 ± 1.7 at the last follow-up (*p* < 0.01).

At the 1-year FU, the CT scan showed complete healing of the osteotomies in all cases. No cases of infection, progression of the deformity, proximal or distal junction pathology, or pulling out of screws were recorded up to the final FU.

## Discussion

The presented technique, although technically demanding, resulted in an acceptable average time and blood loss. These results were superior with respect to the main case series dealing with single-level PSO reported in the literature [11], but were equal or inferior to the rare case series available in the literature that deal with two-level PSO adopted for the treatment of anklylosing spondylitis, which involves a first PSO at the thoraco-lumbar junction (T12 or L1) and another one performed at the lumbar caudal level [12–14].

The technique proved to be really efficient at preventing intraoperative neurologic impairment, with no case showing a motor-evoked potential reduction during resections and during corrective manoeuvres with osteotomy closure. This finding is related, in the authors’ opinion, to some technical issues. First, planning the second osteotomy three levels above the deformity apex and adopting temporary rods permits three pairs of screws to be maintained between the two osteotomies, resulting in strong fixation without the risk of sagittal translation of the portion of the spine between the two osteotomies, which represents the major risk factor for neurological impairment in thoracic PSO [11, 15]. Secondarily, respecting the thoracic nerve roots, especially in the apical-level osteotomy (T11 and T12 in all four cases), lowers the risk of spinal cord ischaemia according to Lau et al. [16]. Finally, the adoption of a wide laminectomy at least one level above and one below the osteotomy site proved to be crucial in preventing spinal cord kinking and compression after bony resections and osteotomy closure.

The technique proved to be really efficient at reducing a deformity angle of up to 75%, thus re-establishing a physiological sagittal alignment, with a reduction of the SVA to within 5 cm in all the patients evaluated. This finding may be explained by the characteristics of the analysed cohort, in which the deformity apex was located at the thoraco-lumbar junction. This permits the first osteotomy to be performed at the T12 or T11 level, where the corrective potential of the thoracic PSO is maximal, and the second to be performed at the T7 or T8 level, where the corrective power remains remarkable [15]. The obtained correction rate is quite impressive and is in line with those reported by authors who performed more aggressive corrective procedures, such as vertebral column resection (VCR), which has, up to now, been considered the gold standard in the treatment of fixed kyphotic deformities for which the amount of correction required exceeds the correction capability of a single PSO [17–22]. Differently from the case series dealing with VCR [17–22], we did not observe any major complication in the presented case series.

The absence of significant changes in spinopelvic parameters can be explained by the fact that these patients had a thoraco-lumbar deformity (with a thoracic apex in all cases) and not a lumbosacral deformity. Therefore, the osteotomies and the corrective manoeuvres were performed with the aim to reduce the hyperkyphosis in the thoracic spine, and not to gain lordosis in the lumbosacral junction. Despite that, the GAP score showed a significant improvement, mainly because of the powerful reduction of the global sagittal imbalance that the technique made it possible to achieve.

Regarding the healing at osteotomy sites, the CT scan performed at the 1-year FU demonstrated excellent healing of all six osteotomies. This finding may be explained by the wide bony contact that is achieved with a PSO, unlike a VCR, in which an anterior cage or mesh is required to avoid spinal cord kneeling, thus lowering the biological healing potential of the spine [17–22]. Another explanation for the excellent healing potential of the presented technique is the presence of the rib cage in the thoracic tract. Yang et al. [23] found that thoracic PSOs had a lower non-union rate, resulting in fewer late mechanical complications compared to lumbar PSOs, which was assumed to be because the greater motion of lumbar vertebrae compared to thoracic vertebrae—which are additionally sustained by the rigid rib cage—may contribute to an increased rate of pseudarthrosis and rod rupture in the lumbar spine.

The adoption of simultaneous double PSOs has rarely been described in the literature. Obeid et al. [24] reported the adoption of two adjacent apical PSOs in a single session with a modification to the standard PSO technique, represented by the resection of the discs above each osteotomized vertebra, for the treatment of a thoraco-lumbar fixed severe kyphotic deformity. The described technique, although fascinating, may be related in the authors’ opinion to a high risk of pseudarthrosis at the osteotomy sites, and presents the drawback of not being appliable in cases in which the anterior annulus is calcified or ossified. Most of the case series dealing with simultaneous PSOs refer to patients affected by ankylosing spondylitis treated with two lumbar PSOs or with a lumbar and a thoracic PSO, with excellent correction rates and the avoidance of major complications [12–14]. The adoption of a double simultaneous thoracic PSO has been reported by Lau et al. [16] and O'Shaughnessy [15], but their case series were extremely heterogeneous in terms of patients and deformity characteristics. The case series presented here does not come without limitations. First, its small sample size and the retrospective nature of the study are clear limitations. Secondly, it deals with a heterogeneous cohort of patients in terms of the aetiology of the deformity (congenital and iatrogenic).

In conclusion, although technically demanding, simultaneous two-level non-contiguous thoracic pedicle subtraction osteotomy is a powerful technique which, with its high corrective potential in the sagittal plane alongside its very low complication rate, may be particularly suitable for the treatment of fixed severe hyperkyphosis in adults with the apex located at the thoraco-lumbar junction.

## Data Availability

The datasets used and/or analysed during the current study are available from the corresponding author on reasonable request.
