# Correction: Multilevel non-contiguous thoracic pedicle subtraction osteotomy for fixed rounded hyperkyphotic deformity of the thoraco-lumbar junction with anterior bony fusion: technical note

**DOI:** 10.1186/s10195-022-00679-y

**Published:** 2022-12-22

**Authors:** Cesare Faldini, Francesca Barile, Giovanni Viroli, Marco Manzetti, Giuseppe Geraci, Alberto Ruffilli

**Affiliations:** Department of Biomedical and Neuromotor Science-DIBINEM, University of Bologna, 1st Orthopaedic and Traumatologic Clinic, IRCCS Istituto Ortopedico Rizzoli, Bologna, Italy

**Correction: Journal of Orthopaedics and Traumatology (2022) 23:47** https://doi.org/10.1186/s10195-022-00665-4

Following publication of the original article [1], the authors identified an error in the author names. The given and family names of the authors were erroneously transposed.

The incorrect author names: Faldini Cesare, Barile Francesca, Viroli Giovanni, Manzetti Marco, Geraci Giuseppe and Ruflli Alberto.

The correct author names: Cesare Faldini, Francesca Barile, Giovanni Viroli, Marco Manzetti, Giuseppe Geraci and Alberto Ruffilli.

The author group has been updated above and the original article [1] has been corrected.
